# Millimeter-scale radioluminescent power for electronic sensors

**DOI:** 10.1016/j.isci.2024.111686

**Published:** 2024-12-25

**Authors:** Averal N. Kandala, Sinan Wang, Joseph E. Blecha, Yung-Hua Wang, Rahul K. Lall, Ali M. Niknejad, Youngho Seo, Michael J. Evans, Robert R. Flavell, Henry F. VanBrocklin, Mekhail Anwar

**Affiliations:** 1 Department of Electrical Engineering and Computer Sciences, University of California, Berkeley, Berkeley, CA 94720, USA; 2 Department of Radiology and Biomedical Imaging, University of California, San Francisco, San Francisco, CA 94107, USA; 3 Department of Radiation Oncology, University of California, San Francisco, San Francisco, CA 94158, USA

**Keywords:** Medical device, Sensor, Optical materials, Bioengineering

## Abstract

The storage and generation of electrical energy at the mm-scale is a core roadblock to realizing many untethered miniature systems, including industrial, environmental, and medically implanted sensors. We describe the potential to address the sensor energy requirement in a two-step process by first converting alpha radiation into light, which can then be translated into electrical power through a photovoltaic harvester circuit protected by a clear sealant. Different phosphorescent and scintillating materials were mixed with the alpha-emitter Th-227, and the conversion efficiency of europium-doped yttrium oxide was the highest at around 2%. Measurements of the light generated by this phosphor when combined with Th-227 reveal that over 100 nW of optical power can be expected at volumes around 1 mm^3^ over more than two months. The use of a clear sealant, together with the evaporation of liquid solution following the mixture, can enable safe miniaturization for size-constrained medical and internet-of-things (IoT) sensor applications.

## Highlights

• Optimized phosphor conversion of Th-227 radiation to light for mm-scale sensor power
• Efficiency model for irradiated phosphors
• Europium-doped yttrium oxide exhibited higher efficiency (∼2%) than other phosphors
• Greater than 100 nW of optical power produced over 2 months at mm-scale

## Graphical abstract

**Figure:**
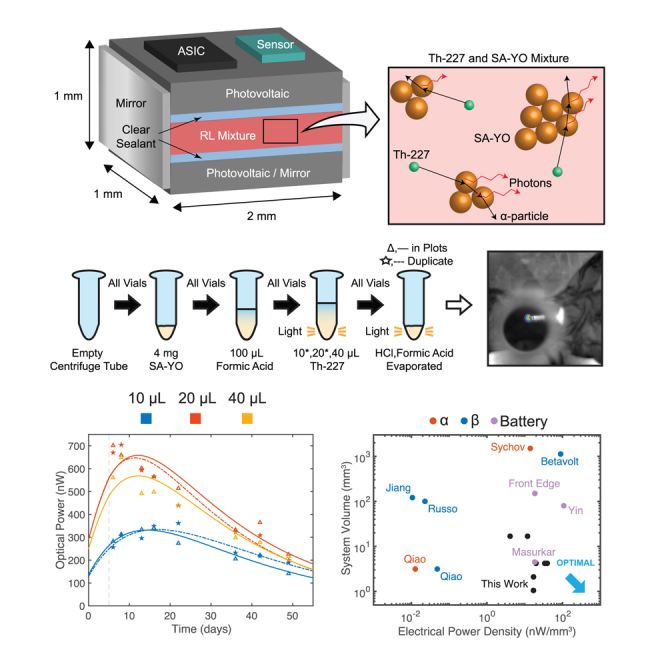

## Introduction

Modern electronic sensing systems operating continuously in isolated, space-constrained environments require a persistent power source that can integrate on a silicon substrate with mm-scale CMOS sensor “chips” while scaling in size to fit the needs of the target application. To achieve overall sensor miniaturization, minimal volume overhead from the power source is desired. This translates to high power density with low package volume. Although lithium batteries have predominated in cm-scale systems, especially those intended for medical applications,^1^ battery technologies face significant fabrication and hermetic packaging challenges that inhibit their application at mm-scale.^2^

To overcome these sizing and scaling challenges, external coupling through ultrasound and electromagnetics has been used to deliver power to smaller, battery-less systems.^3^^,^^4^^,^^5^^,^^6^ However, for many applications, system operation and data collection must continue independent of external tethering. For example, an implanted platform to monitor tumor response during neoadjuvant cancer therapy would require continuous power over at least two months,^7^ setting one benchmark for power source stability. To implement a chip-scale power source capable of months of continuous power output, we evaluate herein highly power-dense alpha-emitting radionuclides and isotopes with low levels of difficult-to-shield gamma, neutron, and bremsstrahlung radiation.^1^

**Figure 1:**
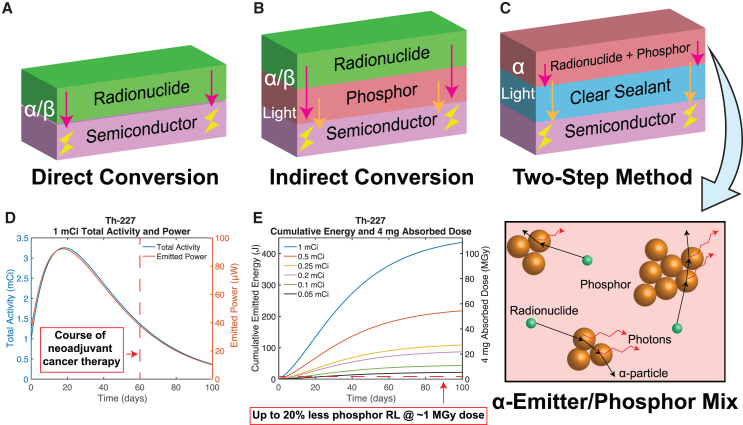
A comparison of compact radiation-to-electricity conversion methods and rationale for the proposed two-step, radioluminescence-based electronic power source (A) A semiconductor substrate can convert incident alpha or beta radiation from an adjacent radionuclide directly into electricity,^1^^,^^8^^,^^10^^,^^11^^,^^12^^,^^13^ promising high efficiency. However, high linear energy transfer can damage the substrate,^8^^,^^11^^,^^13^ limiting longevity, and low penetration depth necessitates structure repetition and sophisticated assembly,^12^ limiting miniaturization. (B) Introduction of a phosphor extends the effective penetration depth of the radiation through an auxiliary conversion to light,^10^^,^^13^^,^^14^^,^^15^^,^^16^^,^^17^ but the conversion efficiency of both the phosphor and substrate can degrade over time due to continued radiation exposure.^13^^,^^14^. (C) In a two-step process, a mixture of a high-energy alpha-emitter with a phosphor ensures maximal conversion of radiation into light for subsequent photovoltaic harvesting by the substrate,^16^ while a clear sealant shields the substrate and surrounding environment from radiation damage.^14^ The inset cartoon illustrates how radiation emitted at any angle can meet a phosphor particle and be converted into light. (D) The total activity and emitted power over time of an initial 1 mCi sample of the alpha-emitting radionuclide Th-227, demonstrating peak power within the critical treatment window for neoadjuvant cancer therapy and suitability for use in a cancer-tracking implant system^7^ (Equations 9 and 10 in Method details). (E) Dose absorbed in 4 mg of phosphor and cumulative energy emitted over time from Th-227 of varying initial activity, mixed with the phosphor at t=0. The high dose and stratification according to initial activity suggest similar stratification in phosphor radioluminescence (RL) efficiency, with just ∼1 MGy dose shown to result in up to 20% lower phosphor RL efficiency^14^ (Equation 16 through Equation 19 in Method details).

Existing strategies to harvest energy from alpha radionuclides suffer from poor longevity and challenges in miniaturization to the mm scale. Thermoelectric generation from heat due to alpha radiation has resulted in cm-scale power sources, but efficiencies between 0.3 and 0.75%^8^ and a need for thermal insulation hinder further miniaturization of this approach.^1^^,^^8^^,^^9^ Direct conversion of alpha or beta radiation into electricity by a semiconductor substrate has the potential to miniaturize form factor with high efficiency^8^^,^^10^^,^^11^^,^^12^ (initially above 25%^10^^,^^12^) (Figure 1A). However, high linear energy transfer from alpha particles damages the substrate, severely degrading its efficiency and longevity to days or hours,^8^^,^^11^^,^^13^ and low penetration depth necessitates specialized stacked structures for sufficient power output, complicating assembly and miniaturization.^12^

Introducing a phosphor between the radionuclide and the substrate to convert the radiation into light improves substrate longevity (Figure 1B), since most of the energy from radionuclide decay is deposited in the phosphor layer.^10^^,^^13^^,^^14^^,^^15^^,^^16^^,^^17^ Here, we propose a two-step conversion method that builds upon this idea but isolates the radioluminescent (RL, radiation-to-light) and photovoltaic (PV, light-to-electricity) conversion steps: first, mix the phosphor and radionuclide together to ensure the radiation has maximal incidence on the phosphor for optimal RL efficiency; then, add a clear sealing layer to pass generated light to the substrate and block radiation, preserving PV efficiency and longevity (Figure 1C). In practice, a mm-scale RL mixture volume could be enclosed by a clear material and then placed on the harvesting photovoltaic section of a sensor chip, minimizing integration overhead.

In this work, we select the high energy alpha-emitter Th-227 as the radionuclide for our system, as it requires minimal shielding and its decay chain includes multiple alpha emissions, enabling power delivery for two months with a delayed power peak ∼20 days after production^18^^,^^19^^,^^20^ (Figure 1D; Method details). This power peak enables frequent and/or higher-power sensor operation in the critical period soon after device assembly and deployment, with high Th-227 cumulative energy output coming at the cost of phosphor RL efficiency degradation over time^14^ (Figure 1E; Method details).

We aim to produce greater than 100 nW of optical power in an mm-scale form factor, enabling reliable energy harvesting in standard silicon even with photovoltaic efficiency below the theoretical maximum of ∼29%.^21^^,^^22^ To meet this goal, we optimize the optical power output and volume of the RL mixture in three steps. First, we select the optimal phosphor for the mixture empirically, measuring light output over time for different phosphors combined with the same amount of Th-227. Then, we vary the amount of the optimal phosphor combined with a fixed amount of Th-227, and vice versa, to identify volume and activity scaling trends for efficiency, optical power, and optical power density. Finally, we investigate the existence of an optical power maximum for a fixed phosphor volume at high Th-227 activity levels.

## Results and discussion

To identify the most efficient material for converting the Th-227 radiation into light, phosphors, and scintillators were procured from commercial vendors, with quantum (light-to-light) yield used as a heuristic indicator of radiation-to-light conversion efficiency during procurement (Table 1).

**Table 1:** Phosphors and scintillators for study

*Material Name*	*Experimental Identifier*	*Supplier*	*Quantum Yield*	*Particle Size*	*Emission Wavelength*
Y_1.92_Eu_0.08_O_3_ (YEO)^23^^,^^24^	SA-YO	Sigma-Aldrich	∼100%	4-8 μm	611 nm
YYG 560 200 Isiphor®^25^	YYG 560	Sigma-Aldrich	>90%	19.5–21.5 μm (D50)	560 nm
SGA 555 100 Isiphor®^26^	SGA 555	Sigma-Aldrich	>90%	12-14 μm (D50)	555 nm
NaY_0.77_Yb_0.20_Er_0__.__03_F_4_^27^^,^^28^	SA-UCPh	Sigma-Aldrich	∼3%	1-5 μm (D50)	940-980 nm
Solid Form CdSSe-ZnS Core/Shell QDs^29^	QSP-645	Ocean NanoTech	>50%	ND	645 nm
Rare Earth Doped Phosphor Nanoparticles^30^	SA-620	Sigma-Aldrich	ND	10 nm	620 nm

D50 signifies that the indicated size approximately reflects the median value.

**Figure 2:**
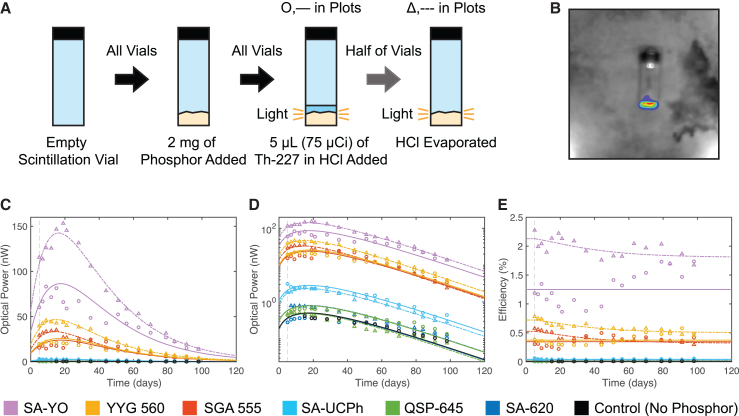
Phosphor comparison process and data Time axes are marked in days since the production and initial assay of the Th-227. The vertical dashed line marks the combination of the Th-227 and phosphors on day 5. CLLSE fits track phosphor RL efficiency degradation after the introduction of Th-227 (Method details). (A) Glass scintillation vials with flat bottoms were filled with 2 mg of each phosphor, before 5 μL of initially 75 μCi Th-227 in HCl solution was introduced. The solution was evaporated from half of these vials in a fume hood, leaving one sample without the solution (evaporated) and one sample with the solution (unevaporated) for each phosphor. The control included 5 μL of unevaporated Th-227 solution without a phosphor. (B) IVIS photograph and luminescence image for evaporated YYG 560 sample on day 15 after Th-227 assay. (C–E) Linear (C) and logarithmic (D) scale optical power and point-by-point RL efficiency (E) time series scatterplots with CLLSE fits. Circles and solid curves designate unevaporated samples, while triangles and dashed curves designate evaporated samples.

**Figure 3:**
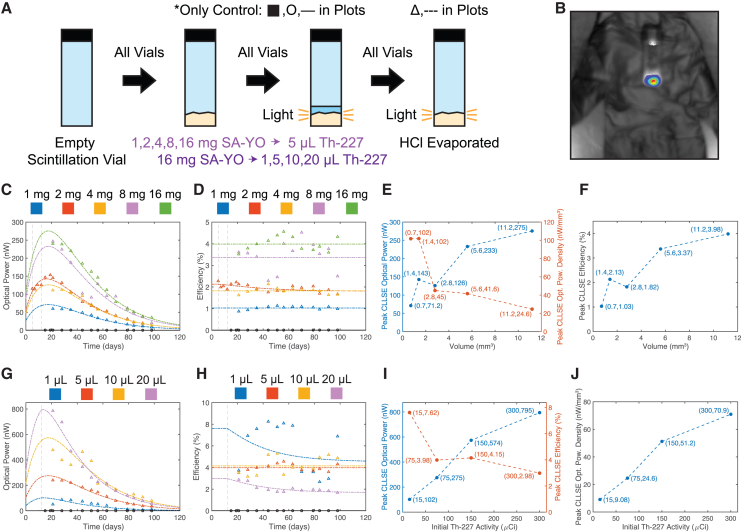
SA-YO and Th-227 sweep process and data Time axes are marked in days since the production and initial assay of the Th-227. Vertical dashed lines mark the combination of the Th-227 and SA-YO on days 5 (2 mg sample from phosphor selection experiment) and 12 (new samples). CLLSE fits track phosphor RL efficiency degradation after the introduction of Th-227 (Method Details). In time series plots (C, D, G, H), circles and solid curves designate unevaporated samples (only the control for this sweep), while triangles and dashed curves designate evaporated samples. (A) For the volume scaling, glass scintillation vials with flat bottoms were filled with 1, 2, 4, 8, or 16 mg of SA-YO (measured concentration of 0.7 mm^3^/mg), before 5 μL of Th-227 in initially 15 μCi/μL HCl solution was introduced. For the activity scaling, vials were filled with 16 mg of SA-YO phosphor, before 1, 5, 10, or 20 μL of the same Th-227 solution was introduced. The solution was evaporated from all phosphor-containing vials in a fume hood. The control (∗) included 5 μL of unevaporated Th-227 solution without a phosphor. (B) IVIS photograph and luminescence image for 16 mg, 5 μL sample on day 56 after Th-227 assay. (C and D) Volume scaling linear scale optical power (C) and point-by-point RL efficiency (D) time series scatterplots with CLLSE fits. (E and F) Volume scaling peak CLLSE optical power (E), optical power density (E), and RL efficiency (F). Increased phosphor volume raises optical power and RL efficiency while lowering optical power density; points are connected with dashed lines to illustrate these trends. (G and H) Activity scaling linear scale optical power (G) and point-by-point RL efficiency (H) time series scatterplots with CLLSE fits. (I and J) Activity scaling peak CLLSE optical power (I), RL efficiency (I), and optical power density (J). Increased initial Th-227 activity raises optical power and optical power density while lowering RL efficiency; points are connected with dashed lines to illustrate these trends.

**Figure 4:**
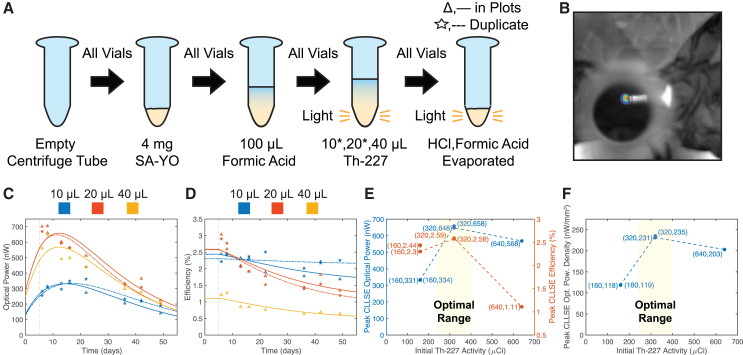
High activity Th-227 sweep process and data Time axes are marked in days since the production and initial assay of the Th-227. A vertical dashed line marks the combination of the Th-227 and SA-YO on day 5. CLLSE fits track phosphor RL efficiency degradation after the introduction of Th-227 (Method details). In time series plots (C, D), stars and dashed curves designate duplicate samples, prepared in addition to identical samples designated by triangles and solid curves. (A) Polypropylene centrifuge tubes with conical bottoms were filled with 4 mg (2.8 mm^3^) of SA-YO before 100 μL of formic acid solution was introduced to promote phosphor interspersion. Then, 10, 20, or 40 μL of Th-227, 16 μCi/μL, in HCl solution was added. Asterisks (∗) indicate the preparation of one duplicate sample. Finally, the solution was evaporated from all samples in a fume hood. (B) IVIS photograph and luminescence image for first 20 μL sample on day 8 after Th-227 assay. To avoid saturation of the camera, a neutral optical density filter was placed above the brightest samples (Supplemental Information). (C and D) Activity scaling linear scale optical power (C) and point-by-point RL efficiency (D) time series scatterplots with CLLSE fits. Over time, RL efficiency becomes stratified according to initial Th-227 activity, with lower activity samples exhibiting higher RL efficiency. (E and F) Activity scaling peak CLLSE optical power (E), RL efficiency (E), and optical power density (F). Points are connected with dashed lines to illustrate trends, and the optimal initial activity range for maximum optical power and optical power density is highlighted.

A Xenogen IVIS 50 Imaging System (“IVIS”) was used to image all Th-227 samples in this study (Supplemental Information). Each material included in the study was confirmed using the IVIS to not phosphoresce at a level beyond the dark signal of the camera in response to ambient lighting. The sample preparation procedure (Method details) for each experiment is illustrated in Figures 2A, 3A, and 4A. A constrained linear least-squares estimation (CLLSE) analysis was developed to fit experimental data (Method details), accounting for phosphor RL efficiency degradation due to cumulative radiation exposure.

### Phosphor selection

Figures 2C–2E demonstrate that vials containing europium-doped yttrium oxide (SA-YO) exhibit the highest light generation for the same Th-227 amount (initial activity of 75 μCi), consistent with the reported high efficiency of this material.^23^ When evaporated, this Th-227/SA-YO combination achieves a peak light output of 140 nW and ∼2% RL efficiency with 1.4 mm^3^ total material volume (0.7 mm^3^/mg), giving a peak power density above 100 nW/mm^3^. The RL efficiency of SA-YO was additionally verified with lower power alpha-emitter Po-210 and beta-emitter Lu-177 (Supplemental Information).

Overall, the phosphors with the highest quantum yield, namely SA-YO, YYG 560, and SGA 555, exhibited the highest empirical RL efficiency as well. The red light (λ = 611 nm)^23^ emission of SA-YO enables efficient harvesting through silicon photovoltaic structures, which have near maximum responsivity in this range, including the IVIS CCD camera (Supplemental Information).^31^ Wavelength compatibility therefore also plays some role in establishing these empirical efficiency values.

The measured optical power and efficiency data generally agree well with the CLLSE curves, confirming that, in each case, radiative power from the Th-227 causes light generation from the phosphor, while also progressively degrading the light-generating capability of the phosphor. The control vial has a minimal level of illumination, most likely due to Cherenkov radiation resulting from the transit of beta particles emitted by Th-227 decay products through the liquid solution.^32^

Unevaporated vials exhibit higher divergence from the model due to possible liquid re-distribution throughout the study and liquid shielding of the phosphor from radiation damage. Variations in vial placement and orientation within the IVIS from measurement time point to time point and the nonuniform distribution of the phosphor and radionuclide within each vial also introduce measurement error. Mixture inhomogeneity due to the flat bottom and non-inclined sides of the scintillation vials exacerbates vial-to-vial uncertainty.

Evaporation was hypothesized to improve radiative energy deposition in the phosphor/scintillator by virtue of removing solvent particles in the medium, which absorb the emitted alpha particle energy, reducing overall efficiency.^16^ However, for QSP-645 and SA-UCPh, which exhibited some of the lowest efficiencies, evaporation resulted in lower light output (Figures 2C and 2D). For each, a lack of thorough mixing between the material and the Th-227 could have limited the RL efficiency. The emission spectrum of SA-UCPh also lies far outside of the efficient conversion range of the IVIS CCD camera (Supplemental Information),^31^ hindering the accurate quantification of its emission.

In general, evaporation yields increased RL efficiency, possibly due to improved radiative energy deposition in the phosphor (Figure 2E), while reducing the mixture volume to just that of the phosphor. Based on these findings, all further samples were evaporated, and SA-YO was selected for further study focused on optimizing light generation with respect to system volume.

### Volume and activity scaling

Next, we analyzed the tradeoffs between light output and volume that come with increasing the amount of SA-YO per unit of Th-227 and vice versa. The SA-YO/Th-227 time series data in Figure 3 show optical power generation on the order of hundreds of nW over the course of two months from a material volume of 5.6 mm^3^ (8 mg of SA-YO). In addition, the sample with the lowest volume of 0.7 mm^3^ (1 mg of SA-YO) produces tens of nW of optical power over the course of three months, illustrating power source scaling below the mm scale.

With increased SA-YO volume, it is likely that fewer emitted alpha particles are “missed” and RL efficiency improves; however, too much SA-YO can lead to photon absorption within the phosphor, eventually limiting RL efficiency gains (Figure 3F). The SA-YO sweep reveals a tradeoff between optical power and optical power density as phosphor volume increases (Figure 3E). Due to reducing gains in RL efficiency and optical power, as a result, optical power density decreases with volume.

Conversely, with higher Th-227 activity, the delivered radiation dose to the phosphor increases, decreasing the efficiency. While optical power and RL efficiency are directly correlated in the phosphor sweep, the Th-227 sweep instead produces a tradeoff between these quantities, as increased input power continues to yield increased output power despite decreasing RL efficiency (Figure 3I). Given the proportional increase in optical power density with Th-227 activity (Figure 3J), the natural next step is to examine whether decreasing RL efficiency eventually produces a maximum in optical power; otherwise, the optimal solution would involve increasing the Th-227 activity as much as possible.

Given the stated goal of miniaturization, low phosphor/mixture volume is a necessity. As a result, thorough intermixture of the phosphor and radionuclide becomes key to maximizing RL efficiency and optical power output. This avoids a scenario in which only a small portion of the phosphor is exposed to most of the radiation, degrading RL efficiency through greatly increased radiation dose and underutilization of the rest of the phosphor.

The disproportionate phosphor volume of 11.2 mm^3^ (16 mg of SA-YO) in the SA-YO sweep, combined with the flat bottoms and non-inclined, phosphor-adhering sides of the glass scintillation vials, made collecting the Th-227 solution and phosphor together very difficult prior to evaporation, especially for the samples with less Th-227 solution. For example, the vial with 1 μL (initially 15 μCi) of the Th-227 solution prior to evaporation contained ∼11 times more phosphor, most of which was not proximal to the deposited Th-227. This incongruity produced high variation in both sweeps for samples with higher phosphor volume and motivated improvements in vial type and mixing procedure in the next phase of the study.

### Power maximization

Fixing the phosphor volume at 2.8 mm^3^ (4 mg of SA-YO), we swept the initial Th-227 activity up to 640 μCi to identify if an optical power maximum exists with respect to the Th-227 amount. To promote a compact and uniform mixture of the phosphor and radionuclide, and thereby maximize RL efficiency, polypropylene centrifuge tubes with conical ends were used as containers for the new samples (Figure 4A; Method Details). In addition, 100 μL of formic acid was added to all samples prior to evaporation to ensure thorough mixing of the Th-227 and SA-YO.

This sweep reveals that an optical power maximum does exist with respect to initial Th-227 activity, as further RL efficiency reductions limit the output power despite increased input power (Figure 4E). An initial activity of 320 μCi yields peak optical power greater than 650 nW and peak optical power density greater than 230 nW/mm^3^, and all samples continue to produce hundreds of nW of optical power over the course of the study.

Measurements of duplicated samples agree well with each other, confirming that the new vial preparation procedure promotes mixture uniformity. In addition, the CLLSE curves fit the observed efficiency trends well (Figure 4D; Method details). As predicted by the model, RL efficiency declines due to the continuous irradiation of the phosphor, with higher activity samples suffering from decreased RL efficiency over time. Further study is required to establish an exact relationship between phosphor radiation dose and RL efficiency.

### Outlook

**Figure 5:**
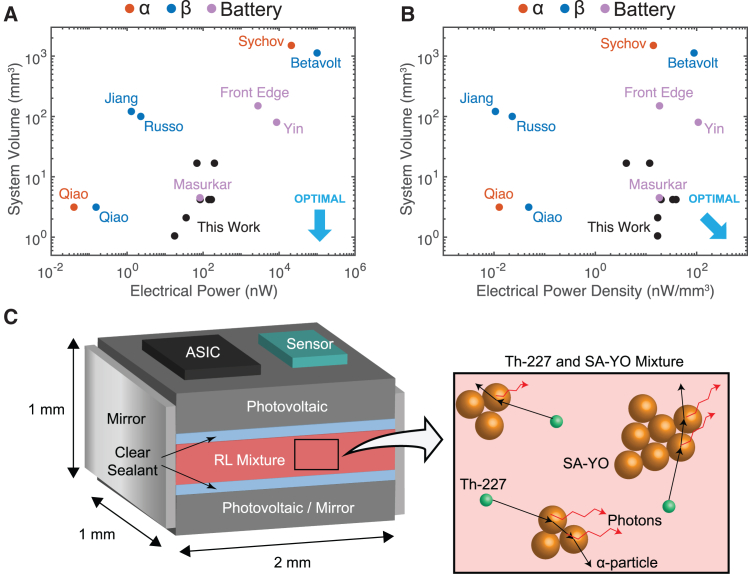
Comparison with prior art and commercially available power sources; mm-scale device concept diagram Designs without an explicit area-to-volume conversion specified are assumed to have a 1 mm minimum total substrate and material thickness. Battery electrical power values are calculated as $V_{mid}C_{d}/\left(60\;\text{days}\right)$, where $V_{mid}$ is average discharge voltage and $C_{d}$ is total discharge capacity, typically specified in Ah. Peak power output is assumed for other radiation-based power sources. For this work, peak power output, 25% PV efficiency, and 50% additional volume overhead from device integration are assumed. (A) Scatterplot comparison in terms of system volume and electrical power. The design goal of minimal volume is achieved toward the bottom of the plot. (B) Scatterplot comparison in terms of system volume and electrical power density. The design goal of minimal volume is achieved toward the bottom right of the plot. (C) mm-scale device concept diagram; components and layers not drawn to exact scale.

The high power production and ease of shielding of radionuclide Th-227, combined with the high efficiency and stability of the phosphor europium-doped yttrium oxide (SA-YO), have the potential to yield a scalable optical power source that can be deposited within a clear sealant and placed directly on a silicon photovoltaic for simple integration with chip-scale sensors. This study shows that optical power in the range of hundreds of nW can be achieved at mm^3^-scale volumes over two months or more through the combination of SA-YO and Th-227. This mixture-based approach lends itself to scaling to sub-mm-scales while still retaining optical power generation on the order of tens of nW, as demonstrated by samples with less phosphor. Assuming 25% PV efficiency and 50% additional volume overhead from device integration,^33^ capabilities exceeding those of power sources demonstrated in research and industry are feasible^14^^,^^16^^,^^17^^,^^34^^,^^35^^,^^36^^,^^37^^,^^38^ (Figures 5A and 5B; Method details).

A conceptual diagram for a radioluminescence-based power module co-fabricated with a photovoltaic array, application-specific integrated circuit (ASIC), and generic sensor is depicted in Figure 5C. One practical approach to complete this integration at the miniature- or macro-scale would involve the placement of a printed circuit board (PCB) containing the photovoltaic array, ASIC, and sensor atop a mirror-coated boat filled with the radioluminescent (RL) mixture. Energy harvesting ASICs using energy collection and duty-cycling strategies have been shown to be capable of efficiently stepping nW of photovoltaic power at low voltage up to 1 V and beyond,^22^ enabling sensing and wireless communication through ultrasonic and electromagnetic means.^5^^,^^39^ While circuit-level scaling challenges, such as the miniaturization of off-chip energy storage capacitors, must be addressed, continuous power over monthslong timescales for mm-scale sensors may be possible.

### Limitations of the study

This work provides empirical validation for an mm-scale radioluminescence (RL) power source, along with an efficiency model based on core radiation dynamics to evaluate continuing phosphor RL degradation due to *in situ* irradiation. However, a theoretical mapping between phosphor absorbed dose and RL efficiency is not established, and further investigation is merited to generalize this work’s conclusions to other phosphors and radionuclides. In addition, a continuation of this study across lower phosphor volumes (corresponding to smaller device sizes) is necessary to demonstrate a reliable scaling trend. Finally, continued research on the packaging of the RL power source, as well as co-fabrication with electronics for sensing, communication, and photovoltaic harvesting, could confirm the applicability of this work to chronic, miniaturized sensing.

## Resource availability

### Lead contact

Requests for further information and resources should be directed to and will be fulfilled by the lead contact, Averal N. Kandala (averal@berkeley.edu), and principal investigator, Mekhail Anwar (mekhail.anwar@ucsf.edu).

### Materials availability

This study did not generate new unique materials.

### Data and code availability

• The data reported in this article will be shared by the lead contact and principal investigator upon request.
• This article does not report the original code.
• Any additional information required to reanalyze the data reported in this article is available from the lead contact and principal investigator upon request.

## Acknowledgments

The authors would like to acknowledge the significant assistance in specimen handling and distribution provided by researchers working in the UCSF China Basin radiation facility, including, but not necessarily limited to, Shalini Chopra, Cyril Fong, Niranjan Meher, Kondapa Naidu Bobba, Ryan Tang, Ning Zhao, and Zhuo Chen. The authors also thank Nirmaan Shanker for his recommendations during article composition.

This material is based upon work supported by the 10.13039/100023581National Science Foundation Graduate Research Fellowship Program under Grant Nos. DGE 1752814 and DGE 2146752. Additional support for this work was provided by philanthropic contributions from the John V. Carbone Jr. Pancreatic Cancer Research Memorial Fund and the Zaidi Family Research Gift Fund.

## Author contributions

**Averal N. Kandala:** Conceptualization, methodology, software, validation, formal analysis, investigation, data curation, writing – original draft, writing – review and editing, visualization, and funding acquisition. **Sinan Wang:** Investigation and resources. **Joseph E. Blecha:** Resources. **Yung-Hua Wang:** Resources. **Rahul K. Lall:** Methodology, validation, formal analysis, and investigation. **Ali M. Niknejad:** Supervision. **Youngho Seo:** Resources, writing – review and editing, and supervision. **Michael J. Evans:** Resources and supervision. **Robert R. Flavell:** Resources and supervision. **Henry F. VanBrocklin:** Resources, writing – review and editing, and supervision. **Mekhail Anwar:** Conceptualization, methodology, writing – review and editing, supervision, project administration, and funding acquisition.

## Declaration of interests

The authors declare no competing interests.

## STAR★Methods

### Key resources table

**Table:** 

REAGENT or RESOURCE	SOURCE	IDENTIFIER
**Chemicals, peptides, and recombinant proteins**		

Yttrium oxide, europium doped (SA-YO)	Sigma-Aldrich	Cat#756490 (https://www.sigmaaldrich.com/catalog/product/aldrich/756490)
YYG 560 200 isiphor ® (YYG 560)	Sigma-Aldrich	Cat#900424 (https://www.sigmaaldrich.com/catalog/product/aldrich/900424)
SGA 555 100 isiphor ® (SGA 555)	Sigma-Aldrich	Cat#900437 (https://www.sigmaaldrich.com/catalog/product/aldrich/900437)
Sodium yttrium fluoride, ytterbium and erbium doped (SA-UCPh)	Sigma-Aldrich	Cat#756555 (https://www.sigmaaldrich.com/catalog/product/aldrich/756555)
CdSSe/ZnS core/shell quantum dot solid (QSP-645)	Ocean NanoTech	Cat#QSP-645 (https://www.oceannanotech.com/by-shipping/nornal-shipping/qpp-645.html)
Rare earth doped phosphor nanoparticles (SA-620)	Sigma-Aldrich	Cat#900557 (https://www.sigmaaldrich.com/catalog/product/aldrich/900557)

**Software and algorithms**		

IVIS Living Image Software	Revvity, PerkinElmer	Cat#128113 (https://www.revvity.com/product/li-software-for-spectrum-1-seat-add-on-128113)
MATLAB	MathWorks	https://www.mathworks.com/products/matlab.html

### Method Details

#### Isotope selection and properties

Th-227 has several important properties that make it an ideal choice for use in a mm-scale power source capable of sustaining months of sensor operation, potentially within the human body. Primarily, its main decay mode is through alpha emissions, which are high energy but easily shielded. Alpha particles also have high linear energy transfer (LET), ensuring maximal energy deposition within the phosphor. Th-227 and its daughter nuclides emit a negligible amount of beta and gamma radiation,^19^ eliminating the need for significant shielding, and greatly reducing the size of the system. As shown in below figure, first daughter Ra-223’s alpha decay is followed by a quick succession of additional decays, providing approximately five times more energy than a single decay alone. This enables a 1 mCi sample of Th-227 to produce more than 30 μW of power over the course of 60 days (Figure 1D), for example. In addition, the transient equilibrium of Th-227 and Ra-223 yields a peak in power approximately 20 days after Th-227 production,^20^ enabling time for power source and device fabrication and integration before deployment. Finally, Th-227 is regularly available for purchase in the United States for authorized research purposes in units of mCi from Oak Ridge National Laboratory (ORNL), enabling experimentation.

**Figure:**
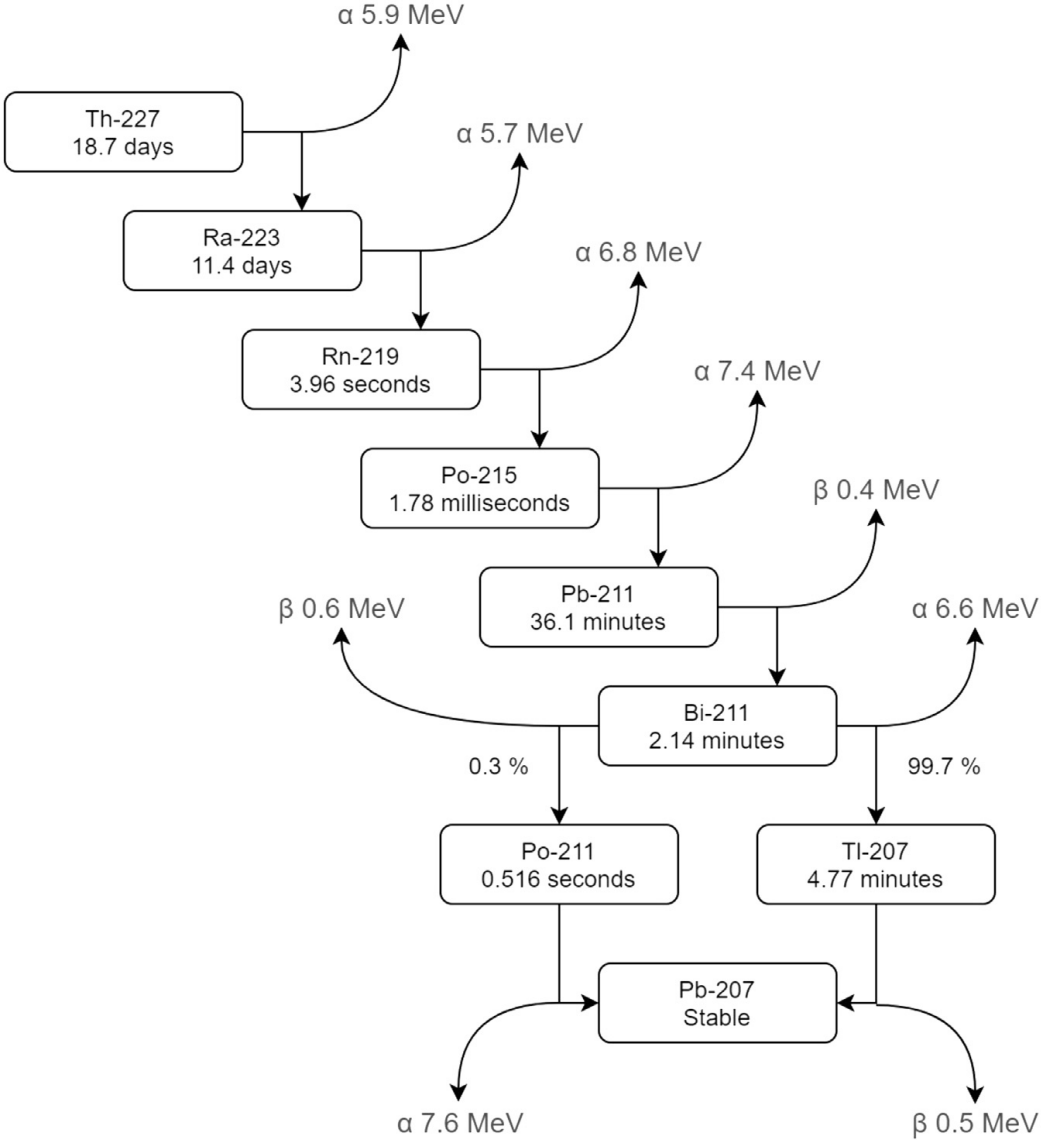
Th-227 decay chain, adapted from Heyerdahl et al.

The following derivation for a system beginning with pure Th-227 details the power dynamics of this decay chain:

(Equation 1) $$\begin{array}{c} \lambda =\frac{\ln\left(2\right)}{T_{1/2}} \end{array}$$

(Equation 2) $$\begin{array}{c} A_{Th}=-\frac{dN_{Th}}{dt}=\lambda_{Th}N_{Th} \end{array}$$

(Equation 3) $$\begin{array}{c} \frac{dN_{Ra}}{dt}=-\lambda_{Ra}N_{Ra}+\lambda_{Th}N_{Th} \end{array}$$

After noting that five additional decays take place rapidly after Ra-223 decays to Rn-219, the system can be approximated as having only a single daughter nuclide by lumping these decays together with the initial Ra-223 decay.

(Equation 4) $$\begin{array}{c} A_{Ra}\approx 6\lambda_{Ra}N_{Ra} \end{array}$$

This, combined with the lack of any initial daughter activity ($A_{Ra,0}=\lambda_{Ra}N_{Ra,0}=0$), yields the following solutions to differential Equations 2 and 3 ^20^:

(Equation 5) $$\begin{array}{c} N_{Th}=N_{Th,0}e^{-\lambda_{Th}t} \end{array}$$

(Equation 6) $$\begin{array}{c} A_{Th}=A_{Th,0}e^{-\lambda_{Th}t} \end{array}$$

(Equation 7) $$\begin{array}{c} N_{Ra}=\frac{\lambda_{Th}}{\lambda_{Ra}-\lambda_{Th}}N_{Th,0}\left(e^{-\lambda_{Th}t}-e^{-\lambda_{Ra}t}\right)+N_{Ra,0}e^{-\lambda_{Ra}t} \end{array}$$

(Equation 8) $$\begin{array}{c} A_{Ra}\approx \frac{{6\lambda }_{Ra}}{\lambda_{Ra}-\lambda_{Th}}A_{Th,0}\left(e^{-\lambda_{Th}t}-e^{-\lambda_{Ra}t}\right) \end{array}$$

(Equation 9) $$\begin{array}{c} A_{tot}\approx A_{Th}+A_{Ra}\approx A_{Th,0}\left(e^{-\lambda_{Th}t}+\frac{{6\lambda }_{Ra}}{\lambda_{Ra}-\lambda_{Th}}\left(e^{-\lambda_{Th}t}-e^{-\lambda_{Ra}t}\right)\right) \end{array}$$

The high energies of the emissions from Th-227 (5.9 MeV^18^) and its daughter nuclides (27.4 MeV, taken altogether and including the contribution of both alpha and beta emissions^18^) allow the total emitted power to be expressed as the following:

(Equation 10) $$\begin{array}{c} P_{tot}\left(t\right)\approx A_{Th,0}\left(5.9\;\text{MeV}\cdot e^{-\lambda_{Th}t}+27.4\;\text{MeV}\cdot \frac{\lambda_{Ra}}{\lambda_{Ra}-\lambda_{Th}}\left(e^{-\lambda_{Th}t}-e^{-\lambda_{Ra}t}\right)\right) \end{array}$$

#### Phosphor RL efficiency model

To model changes in the phosphor RL efficiency over time in response to irradiation, we identify that the differential in the amount of light-producing phosphor should be proportional to the product of the amount of light-producing phosphor, the irradiating power, and the differential in time. $k_{X}$, $k_{Y}$, and $k_{Z}$ are constants and time $t\geq t_{p}$, the time at which the phosphor and radionuclide are combined.

(Equation 11) $$\begin{array}{c} dN_{phosphor}\left(t\right)=k_{X}N_{phosphor}\left(t\right)P_{tot}\left(t\right)dt \end{array}$$

(Equation 12) $$\begin{array}{c} \int_{N_{phosphor}\left(t_{p}\right)}^{N_{phosphor}\left(t\right)}\frac{dN_{phosphor}\left(u\right)}{N_{phosphor}\left(u\right)}=k_{X}\int_{t_{p}}^{t}P_{tot}\left(u\right)du \end{array}$$

(Equation 13) $$\begin{array}{c} \ln\left(N_{phosphor}\left(t\right)\right)-k_{Y}=k_{X}\int_{t_{p}}^{t}P_{tot}\left(u\right)du \end{array}$$

(Equation 14) $$\begin{array}{c} N_{phosphor}\left(t\right)={\exp\;(k_{Y}+k}_{X}\int_{t_{p}}^{t}P_{tot}\left(u\right)du) \end{array}$$

The phosphor RL efficiency is assumed to be proportional to the amount of light-producing phosphor.

(Equation 15) $$\begin{array}{c} \eta \left(t\right)=k_{Z}N_{phosphor}\left(t\right)=k_{Z}{\exp\;(k_{Y}+k}_{X}\int_{t_{p}}^{t}P_{tot}\left(u\right)du) \end{array}$$

The integral of the irradiating power over time is equivalent to the total energy emitted by the Th-227.

(Equation 16) $$\begin{array}{c} E_{tot}\left(t\right)=\int_{0}^{t}P_{tot}\left(u\right)du \end{array}$$

(Equation 17) $$\begin{array}{c} E_{tot}\left(t\right)\approx \frac{A_{Th,0}}{\lambda_{Th}}\left(5.9\;\text{MeV}\cdot \left(1-e^{-\lambda_{Th}t}\right)+27.4\;\text{MeV}\cdot \left(1+\frac{\lambda_{Th}e^{-\lambda_{Ra}t}-\lambda_{Ra}e^{-\lambda_{Th}t}}{\lambda_{Ra}-\lambda_{Th}}\right)\right) \end{array}$$

The cumulative energy delivered to the phosphor is therefore expressed as follows. Assuming the radionuclide is surrounded by the phosphor, the absorbed dose of the phosphor is then that total energy divided by the phosphor mass.

(Equation 18) $$\begin{array}{c} E_{phosphor}\left(t\right)=\int_{t_{p}}^{t}P_{tot}\left(u\right)du=E_{tot}\left(t\right)-E_{tot}\left(t_{p}\right) \end{array}$$

(Equation 19) $$\begin{array}{c} D_{phosphor}\left(t\right)=\frac{E_{phosphor}\left(t\right)}{m} \end{array}$$

Reframing the constant terms $k_{X}$, $k_{Y}$, and $k_{Z}$ as $k_{1}$ and $k_{2}$, and substituting $E_{phosphor}\left(t\right)$ into Equation 15, we arrive at a linear estimation model for the phosphor RL efficiency over time, where $\hat{k_{1}}$ and $\hat{k_{2}}$ are derived empirically through constrained linear least-squares estimation (CLLSE) of η(t) based on measured optical power data and calculations of $E_{phosphor}\left(t\right)$. This hypothesis model is consistent with assumptions and data in prior work.^14^^,^^40^

(Equation 20) $$\begin{array}{c} \;\eta \left(t\right)=k_{1}\;\exp\;\left(k_{2}E_{phosphor}\left(t\right)\right)=\frac{P_{out}\left(t\right)}{P_{tot}\left(t\right)} \end{array}$$

(Equation 21) $$\begin{array}{c} \hat{\eta }\left(t\right)=\hat{k_{1}}\exp\left(\hat{k_{2}}E_{phosphor}\left(t\right)\right),t\geq t_{p} \end{array}$$

(Equation 22) $$\begin{array}{c} e\left(t\right)=\ln\left(\eta \left(t\right)\right)-\ln\left(\hat{\eta }\left(t\right)\right)=\ln\left(\eta \left(t\right)\right)-\ln\left(\hat{k_{1}}\right)-\hat{k_{2}}E_{phosphor}\left(t\right),t\geq t_{p} \end{array}$$

(Equation 23) $$\begin{array}{c} \min_{\hat{k_{1}},\hat{k_{2}}}\;{\left\|e\right\|}_{2}^{2}\;s.t.\;\ln\left(\hat{k_{1}}\;\right)\leq 0,\hat{k_{2}}\leq 0 \end{array}$$

The error time series vector, e, contains samples of e(t), with each sample occurring after time $t_{p}$. The constraints in optimization problem 23 ensure that the estimate of the efficiency begins between 0 and 1 and is monotonically non-increasing and therefore physically feasible. Since irradiation only commences at time $t_{p}$, the efficiency is assumed to be constant before this point, as shown in Equation 24.

(Equation 24) $$\begin{array}{c} \hat{\eta }\left(t\right)=\hat{\eta }\left(t_{p}\right)=\hat{k_{1}},t_{p}\geq t\geq 0 \end{array}$$

(Equation 25) $$\begin{array}{c} \hat{P_{out}}\left(t\right)=\hat{\eta }\left(t\right)\cdot P_{tot}\left(t\right) \end{array}$$

Finally, the CLLSE of the efficiency, defined in Equations 21 and 24, is used to generate an estimate of the phosphor optical power output in Equation 25.

#### Phosphor selection

Table 1 includes the phosphors and scintillators procured for this study, as well as their respective experimental identifiers. As described in Supplemental Information, a Xenogen IVIS 50 Imaging System (“IVIS”) was used to image all Th-227 samples in this study. Each material included in the study was confirmed using the IVIS to not phosphoresce at a level beyond the dark signal of the camera in response to ambient lighting. Th-227 in nitrate form was acquired from ORNL, with 3 mCi dissolved in 200 μL of hydrochloric acid solution (15 μCi/μL). Glass scintillation vials were then prepared containing 2 mg (or the equivalent) of each of the listed materials and approximately 5 μL of this Th-227 solution, yielding an estimated initial activity of 75 μCi for each vial.

In each case, the phosphor/scintillator was deposited and measured within the vial before the radionuclide was inserted via pipette. For each material, an additional identical vial was prepared and then evaporated, leaving two vials containing the same amount of the material and the Th-227 over the course of the experiment: one unevaporated and one evaporated. Evaporation was hypothesized to improve radiative energy deposition in the phosphor/scintillator by virtue of removing superfluous solvent particles in the medium, which absorb the emitted alpha particle energy, reducing overall efficiency.^16^ All vials containing phosphor/scintillator in this portion of the study were prepared on the 5th day following production of the Th-227, with the evaporated SA-620 vial requiring two additional days for evaporation to conclude and measurements to commence. Data from this survey are presented in Figure 2.

As a control, one vial of 5 μL of the original Th-227 solution without any phosphor/scintillator added was prepared 10 days after the samples described above and left unevaporated. Samples were imaged using the IVIS over the course of approximately three months, and these images were converted into total optical power values, covering all 4 π steradians, using the approach described in Supplemental Information. RL efficiency values were calculated according to Equation 20 as the ratio of the total optical power to the total radiative power emitted from the sample, as expressed in Equation 10.

#### Volume and activity scaling

When the combination of the Th-227 solution and the phosphor is evaporated, the volume is dominated by the phosphor. Due to the negligible volume of the radionuclide, the activity can be increased without affecting the system volume. In contrast, increasing the amount of the phosphor directly increases the system volume, while also potentially changing the net RL efficiency due to a tradeoff between having more phosphor to convert the emitted alpha particles to photons and self-absorption of photons within the phosphor.

To investigate system scaling trends and conditions of optimality, samples of varying amounts of SA-YO (measured concentration of 0.7 mm^3^/mg) were prepared on the 12th day following production of the Th-227. Th-227 solution was added to these vials to produce a sweep of the phosphor amount from 1 mg (0.7 mm^3^) to 16 mg (11.2 mm^3^), with the amount of Th-227 solution constant at 5 μL (initially 75 μCi). Next, the Th-227 solution amount was swept from 1 μL (15 μCi) to 20 μL (300 μCi), with the amount of the phosphor constant at 16 mg (11.2 mm^3^). Most of the vials in these sweeps were evaporated on the 19th day following production of the Th-227. The samples for these sweeps were imaged in conjunction with the samples prepared for the original phosphor survey, with data presented together in Figure 3 where applicable.

#### Power maximization

The Th-227 scaling study procedure was modified and repeated to investigate lower volume, higher activity samples, and introduce redundancy through duplicate samples. Th-227 with an initial total activity of 4 mCi was dissolved into 250 μL of hydrochloric acid (HCl) solution (16 μCi/μL). Five polypropylene centrifuge tubes were filled with 4 mg (2.8 mm^3^) of SA-YO and 10 μL (160 μCi, x2), 20 μL (320 μCi, x2), and 40 μL (640 μCi), respectively, of the Th-227 solution. Polypropylene centrifuge tubes with conical ends were used in favor of the previous glass scintillation vials with flat ends to ensure maximal mixture compactness and interspersion and avoid phosphor adhesion to vial sidewalls. To further promote interspersion of the Th-227 within the phosphor, 100 μL of formic acid was added to all samples prior to the addition of the Th-227. Formic acid was selected for this purpose as SA-YO dissolved best in it compared to hydrochloric, acetic, and trifluoroacetic acids. Th-227 was introduced to all five samples on the 5th day following production of the Th-227, and all samples were evaporated that same day, with measurements commencing the following day and continuing for seven weeks. Data from this experiment are presented in Figure 4.

#### Comparison with prior work

Figure 5 presents a graphical comparison between the results of this study and prior published and commercially available power sources. Numerical values for these data are provided in See below tables. For the purposes of this comparison, designs without an explicit area to volume conversion specified are assumed to have 1 mm minimum total substrate and material thickness. Battery electrical power values are calculated as $V_{mid}C_{d}/\left(60\;\text{days}\right)$, where $V_{mid}$ is average discharge voltage and $C_{d}$ is total discharge capacity, typically specified in Ah. Peak power output is assumed for other radiation-based power sources. For this work, peak power output, 25% PV efficiency, and 50% additional volume overhead from device integration are assumed.^33^

**Table:** Prior art and commercially available power sources

*Work*	*Source*	*Active Dimensions*	*Electrical Power*	*Electrical Power Density*
Sychov et al. (2008)^14^	α (5.5 MeV), Pu-238	25 mm × 60 mm = 1500 mm^2^	21000 nW	14 nW/mm^2^
Qiao et al. (2011)^34^	α (5.5 MeV), Am-241	3.14 mm^2^	0.0399 nW	0.0127 nW/mm^2^
Qiao et al. (2011)^34^	β (17 keV), Ni-63	3.14 mm^2^	0.1523 nW	0.0485 nW/mm^2^
Russo et al. (2017)^16^	β (17 keV), Ni-63	100 mm^2^	2.29 nW	0.0229 nW/mm^2^
Jiang et al. (2021)^17^	∼β (10 MeV), Electron beam	11 mm × 11 mm × 11.9 mm = 1440 mm^3^	1.29 nW	0.0009 nW/mm^3^
Betavolt (2024)^38^	β (17 keV), Ni-63	15 mm × 15 mm × 5 mm = 1125 mm^3^	100000 nW	88.889 nW/mm^3^
Masurkar et al. (2018)^35^	Battery	3 mm × 3 mm × 0.5 mm = 4.5 mm^3^	82.562 nW	18.347 nW/mm^3^
Yin et al. (2021)^36^	Battery	10 mm × 10 mm × 0.8 mm = 80 mm^3^	8611.1 nW	107.639 nW/mm^3^
Front Edge (2021)^37^	Battery	20 mm × 25 mm × 0.3 mm = 150 mm^3^	2777.8 nW	18.519 nW/mm^3^

Designs without an explicit area to volume conversion specified are assumed to have 1 mm minimum total substrate and material thickness. Battery electrical power values are calculated as $V_{mid}C_{d}/\left(60\;\text{days}\right)$, where $V_{mid}$ is average discharge voltage and $C_{d}$ is total discharge capacity, typically specified in Ah. Peak power output is assumed for radiation-based power sources.

**Table:** Comparison of selected SA-YO/Th-227 samples from this work and associated values

*SA-YO, Th-227*	*Initial Activity*	*Total Volume*	*Peak CLLSE Optical Power*	*Electrical Power*	*Electrical Power Density*
1 mg, 5 μL	75 μCi	0.7 mm^3^ × 1.5 =1.05 mm^3^	71.2 nW	17.8 nW	16.952 nW/mm^3^
2 mg, 5 μL	75 μCi	1.4 mm^3^ × 1.5 =2.1 mm^3^	143 nW	35.75 nW	17.024 nW/mm^3^
16 mg, 5 μL	75 μCi	11.2 mm^3^ × 1.5 =16.8 mm^3^	275 nW	68.75 nW	4.0923 nW/mm^3^
16 mg, 20 μL	300 μCi	11.2 mm^3^ × 1.5 =16.8 mm^3^	795 nW	198.75 nW	11.830 nW/mm^3^
4 mg, 10 μL	160 μCi	2.8 mm^3^ × 1.5 =4.2 mm^3^	334 nW	83.5 nW	19.881 nW/mm^3^
4 mg, 20 μL	320 μCi	2.8 mm^3^ × 1.5 =4.2 mm^3^	658 nW	164.5 nW	39.167 nW/mm^3^
4 mg, 40 μL	640 μCi	2.8 mm^3^ × 1.5 =4.2 mm^3^	568 nW	142 nW	33.810 nW/mm^3^

Peak optical power output, 25% PV efficiency, and 50% additional volume overhead from device integration are assumed for the comparison.^33^
