# Supplementary material for: Millimeter-scale radioluminescent power for electronic sensors
Source: iScience. 2024 Dec 25;28(1):111686. doi: 10.1016/j.isci.2024.111686 (PMC11772980; doi:10.1016/j.isci.2024.111686)
Supplement: Document S1. Figures S1–S7, Data S1, and Methods S2 and S3 [file mmc1.pdf]

iScience, Volume *28*

Supplemental information

Millimeter-scale radioluminescent power for electronic sensors

Averal N. Kandala, Sinan Wang, Joseph E. Blecha, Yung-Hua Wang, Rahul K. Lall, Ali M. Niknejad, Youngho Seo, Michael J. Evans, Robert R. Flavell, Henry F. VanBrocklin, and Mekhail Anwar

Data S1

Data S1: Supplemental figures, related to STAR Methods and Figures 2, 3, 4, and 5.

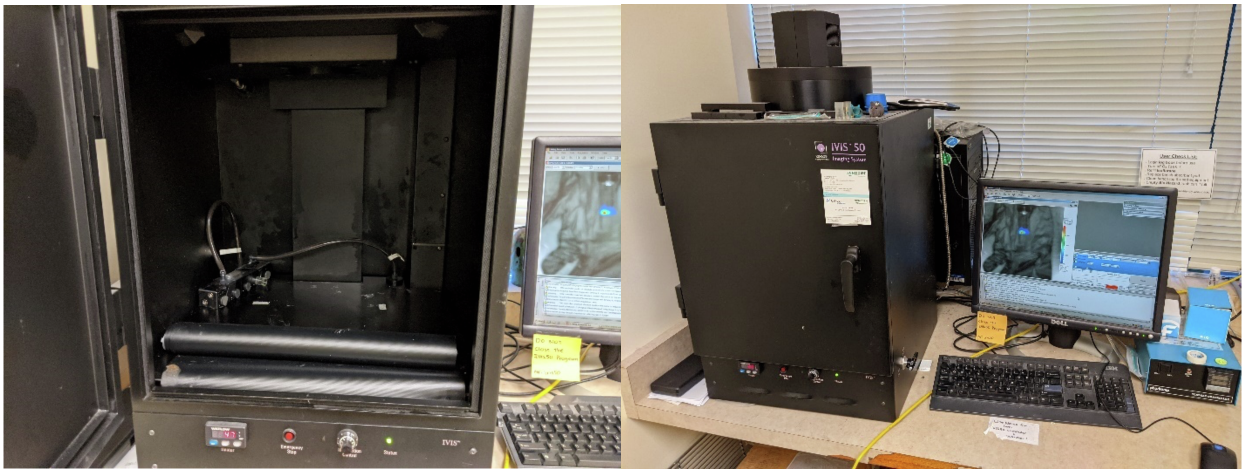

Figure S1 | Xenogen IVIS Imaging System 50 Series used in this study, related to Figures 2, 3, 4, and 5. Left: IVIS interior. Right: IVIS control and computing setup.

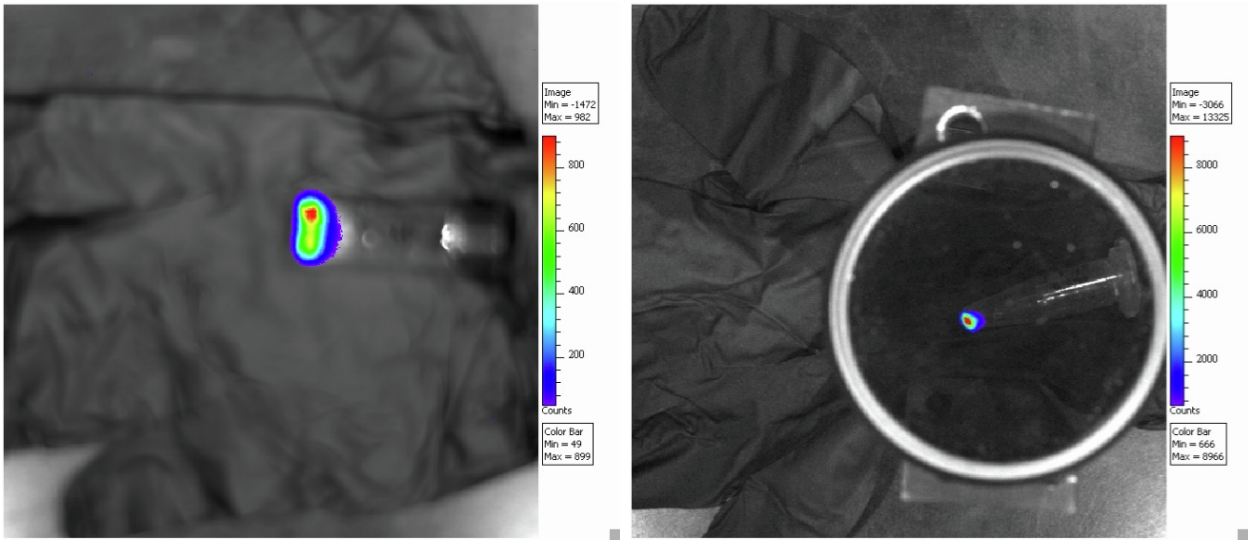

Figure S2 | Xenogen IVIS Imaging System 50 Series images, related to Figures 2, 3, 4, and 5. Left: Relatively unfocused image representative of majority of data points and indicating camera state used for calibration described in Methods S2. Right: Focused image representative of measurements captured near the end of the final experiment, with measurements remaining consistent with prior data.

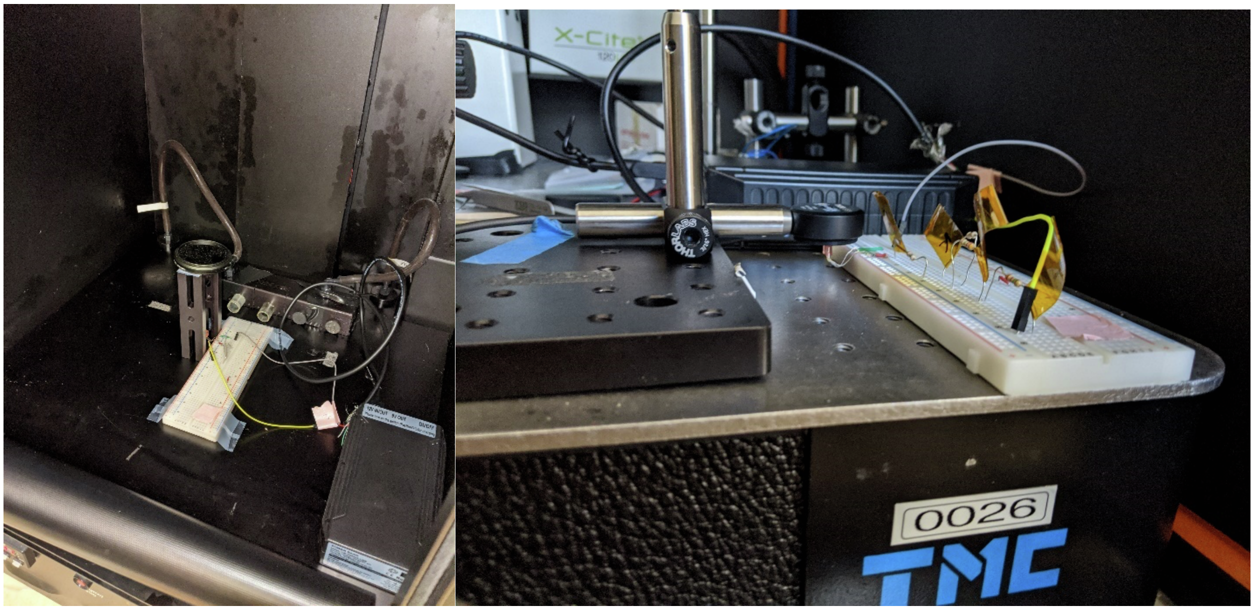

Figure S3 | IVIS calibration process, related to Figures 2, 3, 4, and 5. Left: imaging the calibrating LED within the IVIS using a neutral optical density filter. Right: LED power measurement.

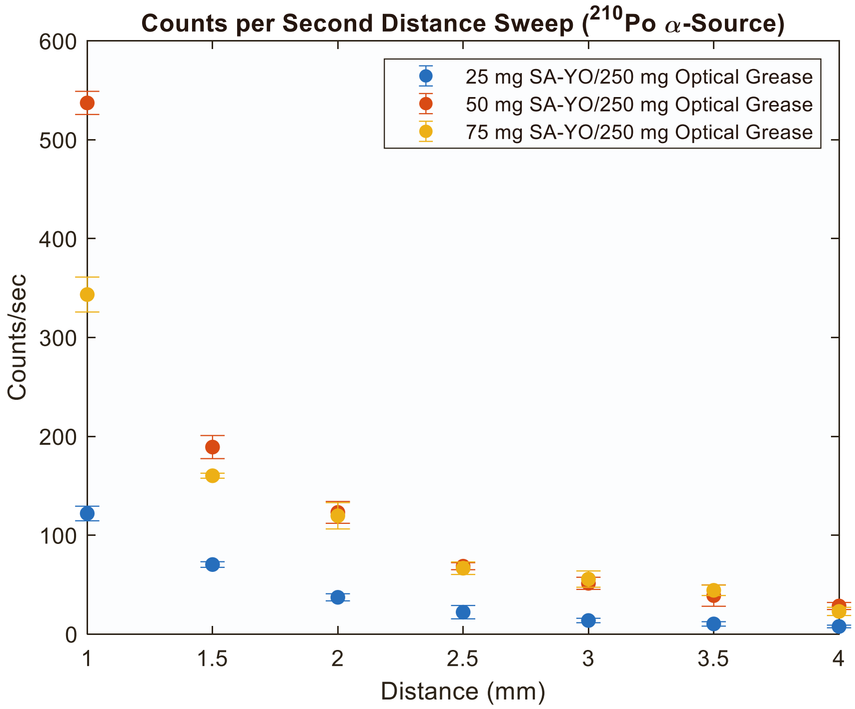

Figure S4 | Single-photon counter raw data, related to STAR Methods. Counts per second were recorded as the scintillating optical fiber was slowly moved away from a 0.1 µCi Po-210 point source in 1 mm increments.

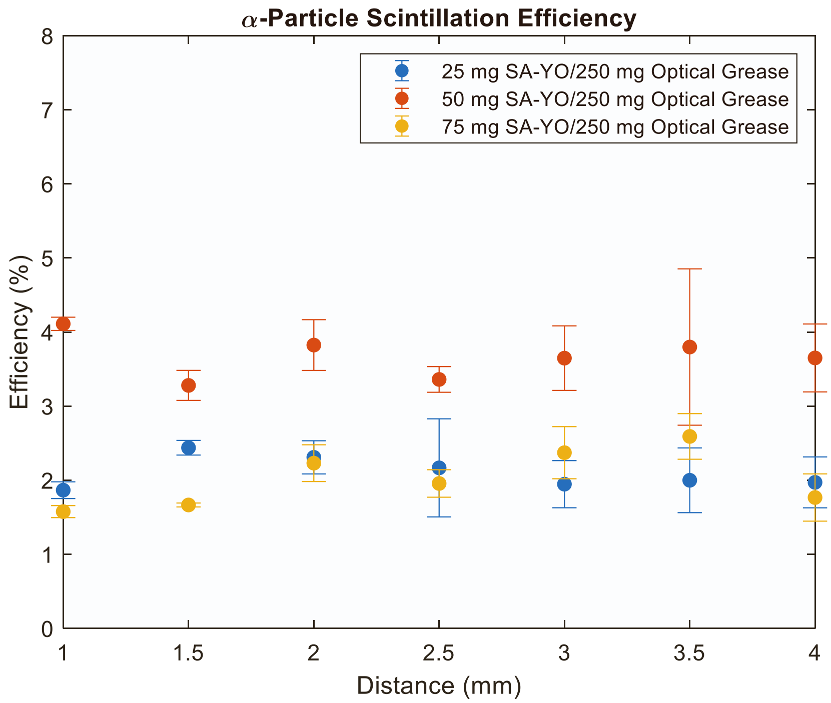

Figure S5 | Scintillation efficiency across distance, related to STAR Methods. The scintillation efficiency at each distance step was evaluated to ensure consistency at all distance increments for all three concentrations of SA-YO in optical grease mixtures.

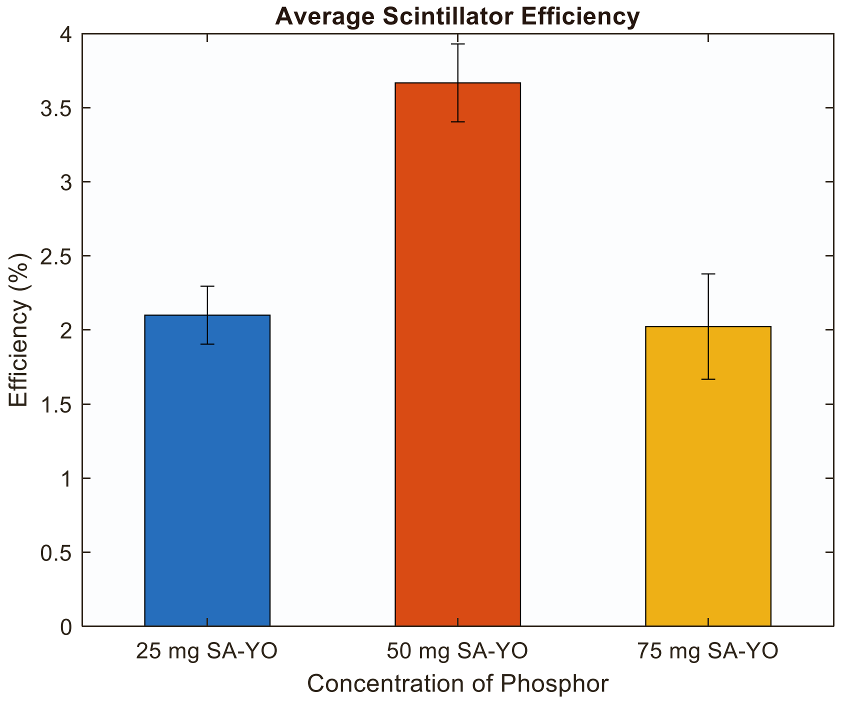

Figure S6 | Average scintillator efficiency, related to STAR Methods. Average scintillation efficiency at different phosphor concentrations of SA-YO in optical grease.

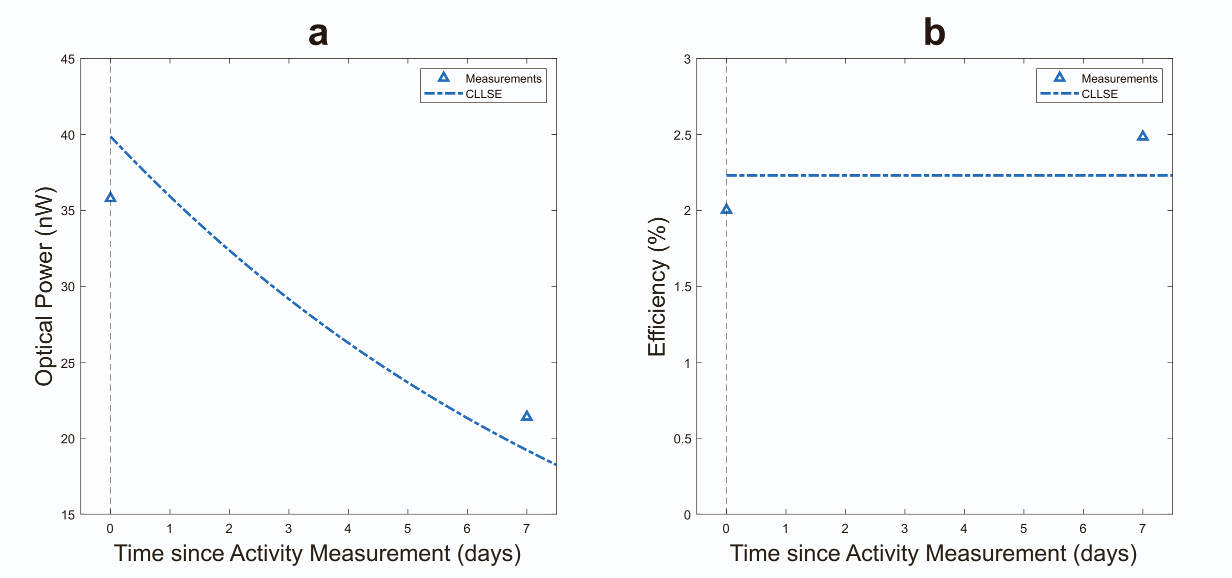

Figure S7 | Lu-177/SA-YO optical power (left) and RL efficiency (right) over brief period, related to STAR Methods. This sample was prepared on the day the Lu-177 was produced, as indicated by the vertical dashed line.

Methods S2

Methods S2: Measurement and setup details, related to STAR Methods and Figures 2, 3, 4, and 5.

Imaging System

A Xenogen IVIS Imaging System 50 Series ("IVIS") machine was used to acquire the precise imaging data presented in this work (Figure S1). The charge-coupling device (CCD) camera of the IVIS is rated to have exceedingly low read noise and ~85% quantum efficiency for light wavelengths between 400 and 700 nm, with this rating dropping to above 50% for all wavelengths between 350 and 900 nm[1]. These properties allowed most materials included in this study to be imaged accurately, with the exception of phosphor identifier SA-UCPh, which emits at infrared wavelengths between 940 and 980 nm[2,3] (Table 1).

However, the values assigned to the pixels of each image produced by this system ("counts") have no nominal conversion to units of optical power specified beyond an undefined proportional relationship with the number of photons incident on the pixels during exposure. As a result, a relatively accurate calibration of the measurements from the IVIS to an optical power reference was necessary, as described in the next section.

Measurement Calibration

The IVIS optical power calibration was achieved using a simple light-emitting diode (LED) circuit, combined with an optical power meter. To form the circuit, a resistor was placed in series with a 5 V DC source and the LED to limit current and set the output optical power. The light from the LED was then measured through the optical power meter within a light-tight box. Finally, the LED was imaged within the IVIS, with a neutral density filter of known transmission covering the LED to avoid camera pixel saturation. This filter was necessary during this calibration process because even the lowest power values that could be read through the optical power meter (tens of nW) would result in image saturation due to the relatively focused nature of the LED light beam.

With values in IVIS "counts" and optical power established for the same light source, a mapping between the two can be found in general, according to the derivation that follows. Note that "total counts" values arise from the summation of all pixel counts of any given image, minus the corresponding background noise values. For example, in the case that multiple subjects are present in the same image, summation would only occur for the pixels representing the subject in question. For images containing only one subject, the summation is done over the entire image, with any extra counts arising from extraneous pixels generally being negligible.

First, the LED has a limited viewing (beam) angle, while the subject (a vial emitting light) emits light uniformly across all 4π steradians, necessitating an analytical calibration.

$$\frac{\text{Imaged Subject Counts}}{\text{Total Subject Counts}} = \frac{\text{IVIS Imaging Solid Angle}}{4\pi} \tag{S1}$$

$$\frac{\text{Imaged LED Counts}}{\text{Total LED Counts}} = \frac{\text{Filter Attenuation} \cdot \text{IVIS Imaging Solid Angle}}{\text{LED Beam Solid Angle}} \tag{S2}$$

$$\frac{\text{Total Subject Power}}{\text{Total LED Power}} = \frac{\text{Total Subject Counts/Subject Exposure Time}}{\text{Totol LED Counts/LED Exposure Time}} \tag{S3}$$

$$P \stackrel{\text{def}}{=} \frac{\text{LED Exposure Time}}{\text{Subject Exposure Time}} \tag{S4}$$

$$\frac{\text{Total Subject Power}}{\text{Total LED Power}} = \frac{P \cdot 4\pi \cdot \text{Filter Attenuation}}{\text{LED Beam Solid Angle}} \cdot \frac{\text{Imaged Subject Counts}}{\text{Imaged LED Counts}} \tag{S5}$$

$$\frac{\text{Total Subject Power}}{\text{Imaged Subject Counts}} = \frac{P \cdot 4\pi \cdot \text{Filter Attenuation}}{\text{LED Beam Solid Angle}} \cdot \frac{\text{Total LED Power}}{\text{Imaged LED Counts}} \tag{S6}$$

Assumptions and Conversion Factor

One core assumption of this derivation is that both the subject and the LED act as point sources of light. This is more accurate for the LED than it is for the subject, as irregularities in material deposition within the test vials, such as phosphor adhesion to vial sidewalls, were observed to yield slightly differing measurements of the same subject, depending on the side viewed. In addition, it is assumed that the LED emits uniformly within the solid angle of its beam. This assumption is mathematically equivalent to assuming linearly degrading optical intensity from the center of the beam and delineating the beam solid angle according to the full-width half-maximum (FWHM).

For further calculations, the viewing angle of the LED was assumed to be 25°, as is common in production, with the half-angle, θ, assumed therefore to be 12.5°. Of note also is the fact that the angle at which the image is collected within the IVIS, the "IVIS Imaging Solid Angle", drops out of the final formula due to the assumption that it is shared between each subject and the calibrating LED. In practice, effort was taken to ensure that this was the case by centering the subject vials within the camera field of view, as was done with the LED. Finally, the solid angle of the LED beam can be calculated using the viewing half-angle, $\theta = 12.5°$, according to the following formula[4]:

$$\text{LED Beam Solid Angle} = 2\pi(1 - \cos\theta) = 0.1489 \text{ [steradians]} \tag{S7}$$

Substituting this value, as well as a measured 23 nW for the total LED power, 1/45.43 for the filter attenuation, and 4426288 for the imaged LED counts over 0.5 seconds of exposure, we arrive at the desired conversion rule, shown below. In practice, this conversion was implemented automatically over the many images using post-processing code which can be provided on request.

$$\frac{\text{Total Subject Power}}{\text{Imaged Subject Counts}} = 0.483 \text{ fW/count} \cdot \frac{\text{10 sec.}}{\text{Subject Exposure Time}} \tag{S8}$$

The parameter P has been decomposed into the form shown above to establish a convenient reference for the exposure time. 10 seconds was the value chosen because it allowed thorough and

timely imaging of relatively dim samples. For the brightest subjects, such as the LED during calibration, the exposure time was often scaled down to the minimum value of 0.5 seconds out of a need to avoid pixel saturation, which can lead to severe underestimation of subject light output.

The neutral density filter used for the IVIS calibration was also used for the brightest radioluminescent (RL) samples, and in post-processing for these measurements, counts were scaled up to account for the filter's attenuation. In these cases, image counts were verified to be heavily concentrated around the RL samples, with minimal contribution (< 6%) from pixels outside of the filter. Image data with inconsistent filter settings were not included in this work.

An additional assumption of the analysis presented in this work is that the optical transmission of the vials holding the samples is unity. In reality, the optical transmission of borosilicate glass in the spectral region of interest is approximately 90%, while the optical transmission of polypropylene was found empirically to be similar, if possibly a bit lower. As a result, the presented figures likely represent an undercount of the actual optical power values, ceteris paribus.

Methods S3

Methods S3: SA-YO RL efficiency verification, related to STAR Methods.

Single Photon Counter Po-210 Experiments

To verify the results presented in this work, an additional experiment was prepared in which SA-YO was homogenously mixed into optical grease (EJ-550, Eljen Technology) and pasted at the tip of a bare optical fiber connected to an avalanche photodiode-based single photon counter (SPCM-AQ4C, Excelitas Technologies).

Three homogenous scintillating pastes were prepared by individually mixing 25 mg, 50 mg, and 75 mg of SA-YO into 250 mg of optical grease. These scintillating pastes were each screen-printed to a thickness of 100 µm. The 100 µm diameter, optical fiber core was then stripped from the cladding of each fiber, leaving 0.5 mm of the core exposed. 100 µm thick films of each scintillating paste were formed at the tip of each fiber by using a clamped precision stage to descend the fiber into its appropriate scintillating paste.

The avalanche photodiodes used in this experiment have their peak quantum efficiency of 58% near the maximum emission peak of SA-YO (610 nm), making this single-photon counter well-equipped for evaluating the efficiency of the phosphor. In addition, the optical transmission of the optical grease at a thickness of 100 µm at this wavelength is approximately 90%, similar to the vials used in the primary experiment.

Once the fiber was coupled to SA-YO, it was clamped facing vertically downwards. A 0.1 µCi Po-210 source was then placed on a precision z-stage, directly underneath the fiber. The z-stage was moved in 0.5 mm increments, sweeping the distance between the Po-210 source and the fiber tip from 1 mm to 5 mm. The whole apparatus was placed in a light-tight optical enclosure (Thorlabs XE25C9) to prevent counts from optical sources other than emitted photons from SA-YO. The counts per second (CPS) were recorded over a 10-minute period at each distance step. The average CPS were computed and plotted as a function of distance (Figure S4). These raw CPS are translated into efficiency by utilizing equations (S9)-(S16).

The input energy to the phosphor can be calculated by first computing the number of alpha particles reaching the tip of the optical fiber core per second (N_α). This can be approximated by finding the solid angle (Ω) of the fiber cross-sectional area with respect to the Po-210 source using the apex half-angle (θ) of the bounding cone of Ω, as seen in equation (S9). The activity of the Po-210 source, A_{Po-210}, is 0.1 µCi. The N_α alpha particles reaching this area per second can then be computed using equation (S10).

$$\Omega = 2\pi(1 - cos\theta) \tag{S9}$$

$$N_\alpha = \frac{A_{Po-210}\Omega}{4\pi} = \frac{(0.1\mu Ci)\left(37000\frac{Bq}{\mu Ci}\right)\Omega}{4\pi} \tag{S10}$$

Po-210 decays primarily via a 5.407 MeV alpha emission (>99.9%), such that all 0.1 μCi can be attributed to alpha decay. The alpha particles emitted from this point source lose energy in air before reaching the detector. The rate of energy loss per unit distance can be approximated as linear over the 0 mm to 5 mm range of interest because the stopping power ($\frac{-dE_\alpha}{dr}$) of a 5.407 MeV ($E_{\alpha,emit}$) alpha particle in air is very constant across this range. Because of the large linear energy transfer (LET) of alpha particles, once in contact with the mixture of SA-YO and optical grease, all of their incident energy will be transferred to this mixture. Therefore, the mass-fraction (*mf*) of SA-YO in the scintillation paste must be considered because energy deposited in the optical grease alone will not result in scintillated light. Given these considerations, the input energy per second can then be calculated from N_α using equation (S10).

$$E_{in} = (E_{\alpha,emit} - \frac{-dE_\alpha}{dr} \cdot r) \cdot mf \cdot N_\alpha \quad \text{(S11)}$$

The output energy of the phosphor can be directly calculated from the measured CPS. Because the scintillating paste is on the tip of the optical fiber, only the incoming light that is totally internally reflected reaches the avalanche photodiodes. This can be calculated by finding the solid angle of totally internally reflected photons as seen in equations (S12) through (S14). This solid angle can then be used to adjust the measured CPS to the actual number of photons emitted by the scintillator per second. The total output energy per second is then computed by multiplying the energy per emitted photon by the number of photons emitted per second (equation (S15)). The phosphor efficiency is given by equation (S16).

$$\gamma_{scint} = \sin^{-1}(NA \cdot n_{scint}/n_{core}) \quad \text{(S12)}$$

$$\Omega_{core} = 2\pi\big(1 - cos(\gamma_{scint})\big) \quad \text{(S13)}$$

$$N_{photons} = \frac{4\pi}{\Omega_{core}}(CPS) \quad \text{(S14)}$$

$$E_{out} = h_{ev}f = 4.1357 \times 10^{-15}\ eV \cdot s \left(\frac{3{\cdot}10^8 \frac{m}{s}}{610\ nm}\right) \quad \text{(S15)}$$

$$\eta = \frac{E_{out}}{E_{in}} \cdot 100 \quad \text{(S16)}$$

The efficiency of the phosphor at each of these distances is shown in Figure S5. Because the distance between the source and the fiber is accounted for, the efficiency of the phosphor at each distance step is expected to be relatively constant. The average efficiency of each of the mixtures can be seen in Figure S6 and varies from 2% to 3.67%. This is in agreement with the primary experimental results discussed earlier. It can be seen that the efficiency peaks at 3.8% for 50 mg of SA-YO and begins to drop soon thereafter. This is a limitation of the experimental setup. Since the alpha particles are incident on one side of the scintillating paste, as the concentration of SA-YO increases the amount of light able to penetrate the paste and be totally internally reflected by the fiber begins to decrease, leading to a drop in scintillation efficiency. In addition, because the

attenuation coefficient of the core without the cladding is higher than with the cladding, this is a slightly conservative estimate of the efficiency.

Lu-177 Experiment

One further experiment in which 4 mg of SA-YO was combined with Lu-177 solution of initial activity 725 μCi prior to evaporation indicated a phosphor RL efficiency of approximately 2%, with typical optical power values on the order of tens of nW (Figure S7). As Lu-177 is a beta-emitter, this confirms that SA-YO exhibits about the same RL efficiency in converting beta radiation into light as it does in converting alpha radiation into light. Due to the inherently low power of beta emissions, Th-227 remained the focal radionuclide of this study.

Supplemental Nomenclature

Ω: Solid angle

NA: Numerical aperture of optical fiber

mf: Fraction of scintillator to optical grease by mass

n_{scint}, n_{core}: Refractive index of the scintillator paste and optical fiber core, respectively

γ_{scint}: Maximum refracted acceptance angle of optical fiber to scintillated photon

CPS: Counts per second

E: Energy input, output, or emitted

η: Efficiency

A_{Po-210}: Activity of Po-210 disk source

r: Distance between radiation source and detector

θ: Apex half-angle of bounding cone of solid angle

N: Number of particles

P: Calibration factor

Supplemental References

1. IVIS 50 Hardware Manual. (2002).
2. Arnaoutakis, G. Characterisation of Up-conversion in Rare-earth Materials. *Edinburgh Instruments Application Notes* 6.
3. Sodium yttrium fluoride, ytterbium and erbium doped 756555. *Sigma-Aldrich* https://www.sigmaaldrich.com/catalog/product/aldrich/756555.
4. Solid angle. *Wikipedia* (2021).
